# Supplementary material for: Murine precursors to type 1 conventional dendritic cells induce tumor cytotoxicity and exhibit activated PD-1/PD-L1 pathway
Source: PLoS One. 2022 Aug 18;17(8):e0273075. doi: 10.1371/journal.pone.0273075 (PMC9387840; doi:10.1371/journal.pone.0273075)
Supplement: Attachment — Submitted filename: Response to Reviewers.docx [file pone.0273075.s001.docx]

| Department of Pediatrics  Hematology/Oncology  College of Medicine | The University of  Arizona_®_  Health Sciences Center   | 1501 N. Campbell Ave.  PO Box 245073  Tucson, Arizona 85724  (520) 626-7053  FAX: (520) 626-6986 |
| --- | --- | --- |

May 10^th^, 2022

Editorial Board

PLOS ONE

Editorial office

Dear Dr. Palaniyandi,

Please find attached our revised manuscript entitled “Murine Precursors to Type 1 Conventional Dendritic Cells Induce Tumor Cytotoxicity and Exhibit Activated PD-1/PD-L1 Pathway” submitted to PLOS ONE (PONE-D-21-26682). We are very pleased that our manuscript was deemed acceptable upon completion of minor revisions requested by one of the two reviewers. We thank the reviewer for their thoughtful comments which have been addressed as detailed below and in the revised manuscript. All changes to the manuscript text are highlighted by track changes.

Journal Requirements:

A number of journal-specific requirements regarding manuscript style and format, data availability, and details on animal research methods and ethics were addressed in our manuscript. These changes are detailed below:

1. We have edited our manuscript to meet PLOS ONE’s style requirements, per the PLOS ONE formatting guide provided by the editor.
2. Our Methods section, subsection “Mice”, was revised to include details on the method of sacrifice (Line 106-108).
3. Our RNA sequencing data set has been uploaded to a public repository in compliance with PLOS ONE’s guidelines. RNA sequencing data have been deposited to the UA Research Data Repository reDATA and are publicly available at the following DOI: <https://doi.org/10.25422/azu.data.14241902>
4. Our manuscript does not contain any blot or gel results that pertain to PLOS ONE’s policy on blot/gel reporting and figure preparation.
5. In regard to PLOS ONE’s data availability policy:
   a. There are no ethical or legal restrictions on sharing the data sets used within our manuscript.
   b. RNA sequencing data have been deposited to the UA Research Data Repository reDATA and are publicly available at the following DOI: <https://doi.org/10.25422/azu.data.14241902>
6. We have removed all instances of “data not shown” within our manuscript (Line 292 and Line 308) to comply with PLOS ONE’s policies on data accessibility, as these data were deemed impertinent to the conclusions drawn from our study.
7. We have included our full ethics statement within the Methods section (Lines 96-100).

Reviewer Comments:

Reviewer #1 stated that the *in vitro* data follows the logic of our manuscript, however there are no *in vivo* data suggest that our *in vitro* findings are relevant to what happens *in vivo*. They further state that our MLR and tumor-killing assays are not necessarily representative of what happens *in vivo* and that without further *in vivo* analysis, our data are not definitive enough for the conclusions drawn.

1. We agree with Reviewer #1 that performing *in vivo* experiments would provide more compelling data and better portray the biological impact of pre-cDC1 function compared to that of CD8α+ cDC1s. Unfortunately, we are limited in our ability to perform rigorous investigations of these DC subsets *in vivo*, as noted in our discussion (Lines 639-642). Limiting factors include:
   a. Lack of suitable mouse models: While Batf3 KO mice are a well-established mouse model that lack CD8α+ cDC1s and retain pre-cDC1s, these mice exhibit significant deficiencies and overall changes in the DC compartment. As such, their ability to mount adaptive T-cell responses is severely altered making this mouse system unsuitable for addressing the questions at hand.
   b. Extreme rarity: pre-cDC1s and CD8α+ cDC1s are two extremely rare cell subsets, with <50,000 CD8α+ cDC1s and <20,000 pre-cDC1s isolated from the spleens of naïve mice. These exceedingly low cell numbers make it extremely technically challenging to evaluate pre-cDC1 and CD8α+ cDC1 function *in vivo*. In addition, mouse models of bone marrow transplantation require whole body irradiation and chemotherapy treatment which dramatically reduce lymphoid cell numbers, making it virtually impossible to examine pre-cDC1s and CD8α+ cDC1s through cell isolation or adoptive transfer in this context.

We have emphasized these specific points within our manuscript (Lines 254-257 and Lines 550-555).

2. We additionally have emphasized in our discussion that this work precipitated from previously published findings that found a strong association between the frequency of host pre-cDC1s and a reduction in death from lethal GvHD in a major-mismatched BMT model (Lines 642-645).
3. We have edited our manuscript to indicate that our functional assays are better described as *ex vivo*, as opposed to *in vitro*. This serves to clarify that all dendritic cells and T-cells used in functional assays were primary cells derived directly from living mouse tissue. Primary cells are recognized for closely mimicking *in vivo* cells by retaining the morphological and functional characteristics of their tissue of origin. The experimental use of primary cells therefore generates more relevant data representing living systems.
4. We have also edited our manuscript to appropriately frame our findings within the borders of *ex vivo* experimentation and emphasize that our interpretations are not extrapolated to make conclusions about what occurs *in vivo*.
5. Lastly, we would like to acknowledge that working to develop rigorous methods to study these cells in vivo would provide compelling and important information. However, as the original submission of this manuscript was in August 2021, the lead author (MSM) who performed these experiments as part of her PhD work has since departed from the University of Arizona to complete her post-doctoral training at another institution. Additionally, the second and third authors who assisted in these studies have also moved to other positions. This unfortunately limits our ability to collect further data.

Reviewer #2: Indicated that she/he did not find any issues that needed to be addressed and further complimented that our study provides important new findings in the field and that the manuscript can be accepted and published at its current state. We thank the reviewer for their time and positive remarks.

We thank you and the reviewers for taking the time to review our manuscript. We sincerely hope that our revisions have adequately addressed the minor comments of Reviewer #1. We look forward to hearing back from you soon and hope that our submission can be accepted without requiring additional re-review. As it has been nine months since our initial submission, we are excited to move forward with this publication.

We thank you again for your time and careful consideration of our revised manuscript, and we look forward to your final decision.

Sincerely,

Megan Stanley Molina ([meganm4@email.arizona.edu](mailto:meganm4@email.arizona.edu))

Emely A Hoffman ([hoffmane@email.arizona.edu](mailto:hoffmane@email.arizona.edu))

Jessica Stokes ([stokes@email.arizona.edu](mailto:stokes@email.arizona.edu))

Nicole Kummet ([nkummet@email.arizona.edu](mailto:nkummet@email.arizona.edu))

Richard J Simpson ([rjsimpson@email.arizona.edu](mailto:rjsimpson@email.arizona.edu))

Emmanuel Katsanis ([ekatsanis@peds.arizona.edu](mailto:ekatsanis@peds.arizona.edu))
